# Virtual screening and in silico design of novel inhibitors of bacterial lectins

**DOI:** 10.1186/1758-2946-4-S1-P1

**Published:** 2012-05-01

**Authors:** Jan Alán, Petr Kulhánek, Jaroslav Koča

**Affiliations:** 1 CEITEC – Central European Institute of Technology, Masaryk University, Kamenice 5, 625 00 Brno, Czech Republic; 2 National Centre for Biomolecular Research, Faculty of Science, Masaryk University, Kotláská 2, 611 37 Brno, Czech Republic

## 

Bacterium *Pseudomonas aeruginosa* is a human opportunistic pathogen. It can cause infection of immunocompromised people or people suffering from cystic fibrosis, which is often fatal. Bacterial colonization of human tissues is mediated by interaction of bacterial surface proteins – lectins – with cellular surface carbohydrates. PA-IIL is *Pseudomonas aeruginosa* tetrameric lectin, which contains two calcium ions in each binding site and recognizes fucosylated oligosaccharides [1]. Saturation of bacterial surface lectins by specially designed compounds might prevent adhesion to host tissues and thus suppress the infection. In this work, virtual screening and docking were used for identification of compounds that might inhibit this interaction and be potentially used as a new generation of antibiotics.

Two different approaches for identification of promising compounds were employed. Subset of drug-like molecules was docked into the PA-IIL binding site by Autodock Vina [2] and Dock 6 [3]. Interesting ligands were then selected by identifying those with highest score provided by both programs. In the second approach, we focused on the identification of interesting molecular fragments, which should be attached to already pre-docked carbohydrate. The carbohydrate serves as a targeting agent and newly identified fragments increase its interaction with the lectin. In both approaches, ZINC library [4] was the source of ligands and fragments.
